# Inflammatory and immune responses in the cochlea: potential therapeutic targets for sensorineural hearing loss

**DOI:** 10.3389/fphar.2014.00287

**Published:** 2014-12-23

**Authors:** Masato Fujioka, Hideyuki Okano, Kaoru Ogawa

**Affiliations:** ^1^Department of Otorhinolaryngology, Head and Neck Surgery, School of Medicine, Keio UniversityTokyo, Japan; ^2^Department of Physiology, School of Medicine, Keio UniversityTokyo, Japan

**Keywords:** inner ear, cochlea, hearing loss, inflammation, immune response, cochlear macrophage, perivascular melanocyte-like macrophage (PVM/M), microarray analyses

## Abstract

The inner ear was previously assumed to be an “immune-privileged” organ due to the existence of its tight junction-based blood-labyrinth barrier. However, studies performed during the past decade revealed that the mesenchymal region of the cochlea, including its lateral wall, is a common site of inflammation. Neutrophils do not enter this region, which is consistent with the old dogma; however, bone marrow-derived resident macrophages are always present in the spiral ligament of the lateral wall and are activated in response to various types of insults, including noise exposure, ischemia, mitochondrial damage, and surgical stress. Recent studies have also revealed another type of immune cell, called perivascular melanocyte-like macrophages (PVM/Ms), in the stria vascularis. These dedicated antigen-presenting cells also control vascular contraction and permeability. This review discusses a series of reports regarding inflammatory/immune cells in the cochlear lateral wall, the pathways involved in cochlear damage and their potential as therapeutic targets.

## INTRODUCTION

The cochlea is the end organ of the auditory system and is responsible for transducing sound stimuli into a nerve impulse. Sound from the environment travels as a wave through a medium (e.g., air, water) and is conducted through the ossicular chain of the middle ear; the mechanical vibration of the bones is converted to a compression wave in the inner ear fluid, which vibrates a structure in the cochlea called the organ of Corti. The hair cells at the top of the organ of Corti convert the physical mechanical movement into action potentials through their mechanotransduction channels, and this signal is transferred to the brain via bipolar auditory neurons. Through this process, this spiral-shaped, fluid-filled organ located deep in the temporal bone transmits middle ear mechanics to the central nervous system (CNS; LeMasurier and Gillespie, 2005).

Non-sensory cells in the cochlea are assumed to play an important role in generating the conductance of the cochlea for the electrophysiological activity of hair cells (Hibino and Kurachi, 2006). Differences in ion concentrations between partitions in the cochlea and the endocochlear potential in the endolymph are required for the mechanotransduction channels on the hair cells to generate an action potential. The mesenchymal cells in the spiral ligament of lateral wall, the spiral limbs and the epithelial cells in the stria vascularis are the sources of conductance via the ATP-dependent activity of ion pumps (Hibino and Kurachi, 2006; Guinan et al., 2012). In contrast to the avascular organ of Corti, the lateral wall, and spiral limbs are known for their “vascular rich” environment, and researchers have assumed that the microvessels in the area form an essential source of water and ions. The concept is similar to the situation in the kidney, another organ that is exposed to the outer environment and is responsible for the maintenance of fluid homeostasis: the spaces are separated by tight junctions, and ions are constantly recycled between partitions.

By contrast, the inner ear, including the cochlea, was assumed to be an immune-privileged organ, similar to the eyes or brain, due to the existence of tight junctions (McCabe, 1989). In this context, this tight junction-based structure in the stria vascularis is called the “blood-labyrinth barrier,” and it is believed that the barrier blocks immune cells, inner ear antigens, and antibodies (Harris, 1983, 1984). This concept explains immune-mediated hearing loss or autoimmune inner ear disorders. Due to the barrier, the systemic immune system cannot recognize inner ear antigens, and when these antigens are released due to insult-induced damage to the barrier, the immune system inappropriately mounts an attack on the “foreign” antigen (Harris et al., 1985).

Decades have passed since the dogma of immune privilege was established, and several lines of evidence have revealed two cell types of macrophage lineage in the lateral wall of the cochlea: “cochlear macrophages” in the spiral ligament and “perivascular macrophage-like melanocytes (PVM/Ms)” in the stria vascularis (Zhang et al., 2012). The spiral ligament is indeed a common site of inflammation, and cochlear macrophages are responsible for these inflammatory responses for communication with spiral ligament fibrocytes (Hirose et al., 2005). The stria vascularis is, as mentioned above, the site of tight junctions, and the PVM/Ms are responsible for controlling the permeability of the barrier when inflammatory/immune responses are induced by the local release of inflammatory cytokines or chemokines (Zhang et al., 2012).

This article describes a series of reports from the literature regarding those macrophage-like cells, including reports on their roles in inflammatory/immune responses and the maintenance of cochlear homeostasis. Additionally, this article describes gene expression networks in the lateral wall that are activated in response to damage. Potential uses of those cells and signals as therapeutic targets are also discussed.

## IMMUNE CELLS/INFLAMMATORY CELLS IN THE COCHLEA

### COCHLEAR MACROPHAGES

Overexposure to intense noise is a well-known cause of hearing impairment both clinically and experimentally. After decades of investigations on the detailed pathophysiology of hair cells, researchers began to direct their attention to the phenomena occurring in the non-sensory region during and after noise trauma. Wang and Hirose precisely documented the histopathology of the non-sensory region of the cochlea, including its lateral wall spiral ligament, stria vascularis, and spiral limbs, during noise-induced hearing loss in mice. They reported the loss of fibrocytes, the degeneration of stria cells and the partial recovery of fibrocytes after overexposure to noise (Wang et al., 2002; Hirose and Liberman, 2003).

Early electron microscopy studies of the noise-damaged cochlea reported the presence of inflammatory cells (Fredelius and Rask-Andersen, 1990; Fredelius et al., 1990), but quantitative analyses and close examination of the roles of those cells were not performed due to technological limitations. In the early 2000s, research tools for investigating inflammatory and/or immune responses were extensively developed and were applied in various organs. These tools were not used only in normal tissue; researchers began to investigate lesions in various parts of the body, including the CNS, such as ischemic or traumatized brains (Hu et al., 2014). Antibodies labeling specific linages were used, and numerous genetically engineered knockout or transgenic mice were developed. Protocols for sorting particular lineages of hematopoietic stem cells or bone marrow mesenchymal cells became well-established, and bone marrow transplantation to follow the lineage *in vivo* became popular among immunologists (Hess et al., 2004).

The distribution of immune cells and inflammatory responses in the cochlea after ototoxic insults was examined by several groups. Those reports indicated that the mesenchymal region is a common site of inflammation. Hirose et al. (2005) revealed the infiltration of CD45+ Iba1+ CX3CR1+ cells in the cochlea after noise exposure, and they called the cells “cochlear macrophages.” Lang et al. (2006) showed that the cells were positive for CD11b, CD68, ED1, and PKC-βII and that the cells derived from bone marrow cells. Okano et al. (2008) showed that the number of cochlear macrophages increased after sham surgery and that the turnover time of cochlear macrophages in mice was longer than 6 months. Although the precise roles of cochlear macrophages remain unclear, morphological observations suggest that cochlear macrophages clear dead or dying cells by phagocytosis (Hirose et al., 2005). Specific ablation of a particular lineage at crucial timepoints using a genetically modified mouse would provide a mechanism for determining the role of inflammatory cells.

Other roles have been proposed for the macrophages in the lateral wall spiral ligament. Shi and her colleagues have shown that the macrophages in the type V fibrocyte region, around the junction of Reissner’s membrane and the lateral wall, were coupled to microvessels and contributed to the liquid flow by inducing vasospasms in response to noise insults (Dai and Shi, 2011). The decrease in blood flow in the lateral wall during and after noise exposure has long been known, but the underlying mechanics were not fully elucidated until that work was published. The new findings may produce another future therapeutic target for noise-induced hearing loss.

### PERIVASCULAR MELANOCYTE-LIKE MACROPHAGES

Perivascular resident macrophages (PVMs) are macrophages that exist in many tissues, including the brain and retina (Cuadros and Navascues, 1998; Hess et al., 2004). In each organ, PVMs are closely associated with microvessels and intertwined with endothelial cells, and they express common macrophage markers such as F4/80, CD68, CD11b, and MHC class II (Shi, 2010). The known roles of PVMs include immunologic defense and tissue repair. Individual cells remain in place for a long period, but after damage, new cells are recruited from bone marrow, producing turnover at the site of damage (Hess et al., 2004).

The lateral wall stria vascularis is another spiral-shaped “vascular rich” part of the cochlea. This structure is composed of three layers of cells, marginal cells, intermediate cells and basal cells, and capillaries are distributed between the layers (Hibino and Kurachi, 2006). Tight junctions in the stria vascularis establish compositionally distinct fluid compartments. Homeostasis among the endolymph, perilymph, and circulating serum in the capillaries is maintained properly due to this structure.

The presence of melanocytes in this region has been known for two decades (Masuda et al., 1994), but their role was not fully understood. In the early 2010s, marker studies using antibodies similar to those mentioned above showed the presence of immune cells or inflammatory cells with the features of melanocytes. Again, Shi and her colleagues found that F4/80+ GST+ melanocyte-like macrophages are always present in the perivascular region of the stria vascularis (Zhang et al., 2012). The cells were observed on the surface of endothelial cells in the stria region, suggesting that the immune cells are coupled with microvessels and control the integrity of the blood-labyrinth barrier. Indeed, they demonstrated that the depletion of PVM/Ms achieved by gene targeting produced leaky capillaries and elevated hearing thresholds *in vivo*, demonstrating that these cells are indispensable for the maintenance of cochlear fluid homeostasis (Zhang et al., 2012).

Numerous reports, both experimental and clinical, have shown the occurrence of hydrops in immune-mediated inner ear disorders, but the molecular and cellular mechanisms of hydrops remain to be elucidated. Although there is not yet any direct evidence, the roles of PVM/Ms in the control of fluid exchange with the systemic circulation in response to immune-mediated or inflammatory responses may account for the above-mentioned phenomenon.

## INFLAMMATORY/IMMUNE-RELATED GENE EXPRESSION IN THE DAMAGED COCHLEA

### GENE EXPRESSION PROFILING OF THE LATERAL WALL AFTER COCHLEAR DAMAGE

Omics biology is a powerful approach for obtaining a comprehensive understanding of local phenomena occurring in diseases. Several microarray analyses have been performed in the cochlea after insults. Vazquez and colleagues examined the gene expression profile in the whole mouse cochlea 6 h after noise exposure and showed the upregulation of immune genes including CXCL10, SOCS3, Ifrd1, Ifi202b, Igh-6, and TCl1b1 (Gratton et al., 2011). Wangemann’s group reported the expression of numerous inflammatory/immune-related genes in the stria vascularis and revealed an increase in genes associated with macrophages and dendritic cells, including CD68 and MHC class II genes, in the stria vascularis of Slc26a4 knockout mouse (Jabba et al., 2006). They also showed higher numbers of pigmented cells in the knockout mouse than in the littermate control (Jabba et al., 2006). In addition, we reported increased expression of numerous inflammatory genes in the lateral wall spiral ligament after 3NP-induced acute mitochondrial dysfunction (Fujioka et al., 2014). Collectively, these reports demonstrate the presence of inflammatory/immune cells and their involvement in the cochlear damage.

### INFLAMMATORY CYTOKINES OR CHEMOKINES

In general, proinflammatory cytokines are among the first genes upregulated in response to insults at sites of inflammation. These cytokines are produced in various organs after tissue damage in experimental models of the immune response, infection, ischemia, trauma, cryo-ablation, and burns (Berti et al., 2002; Pier et al., 2004), and they are produced by various cell types, including residential immune-related cells (e.g., leukocytes, macrophages, microglia, dendritic cells) and neurons and glia in the CNS. The signaling downstream from proinflammatory cytokines includes the attraction and activation of immune cells, including leukocytes and macrophages.

Because cytokine signals are transmitted to target cells in an autocrine/paracrine manner, the blockade of signals using an antagonist or antibody preparation can be used as a new therapeutic, as demonstrated by the use of infliximab, etanercept, or tocilizumab in inflammatory diseases (Nishimoto and Kishimoto, 2006; Willrich et al., 2014). Therefore, understanding the behavior and the physiological and pathophysiological roles of inflammatory cytokines is critical for translational research into the diseases of each organ, presumably including the cochlea.

### INFLAMMATORY CYTOKINES OR CHEMOKINES INVOLVED IN IMMUNE-MEDIATED HEARING LOSS

The *in vivo* production of TNF-α, IL-1β, and IL-6 in the cochlea, along with synergic leukocyte infiltration, has been described in experimental studies of inner ear inflammation (Satoh et al., 2003; Hashimoto et al., 2005). In this model, the researchers first sensitized the animals using an intraperitoneal injection of an antigen, keyhole limpet hemocyanin (KLH). Next, they surgically injected KLH into the cochlea. In those animals, an immune response occurred in the cochlea, including the recruitment of many immune cells. In addition, an *in vivo* loss-of-function analysis demonstrated that TNF-α was a factor that worsened cochlear inflammation (Satoh et al., 2003).

These studies are very important because such molecules are in general, well-known for their potential to induce secondary inflammatory responses, including leukocyte infiltration, scar formation, and gliosis, in other injured organs. Based on the results of animal studies, several clinical trials are currently in progress, some of which have preliminary results. Future therapeutics based on these results may become available in the near future, although the translation takes time.

### INFLAMMATORY CYTOKINES INVOLVED IN NOISE-INDUCED HEARING LOSS

The authors’ group first reported that proinflammatory cytokines, TNF-α, IL-1β and IL-6, were produced in cochlear fibrocytes damaged by noise and that the time courses of their expression levels were very similar to those observed in other traumatized organs (Fujioka et al., 2006). The blockage of one of these proinflammatory cytokines, IL-6, drastically reduced the number of Iba-1-positive cochlear macrophages, suggesting that the infiltration and activation of those immune cells occur in response to the cytokine (Wakabayashi et al., 2010). Although the effect of the medication in the noise-damaged mouse was relatively small, of which amelioration in hearing loss was within 20 dB, and could only be observed in the apical area, which is in charge of lower frequency sounds, this result indicates that therapeutics targeting inflammatory cytokines or chemokines show potential.

### INFLAMMATORY CYTOKINES AND CHEMOKINES INVOLVED IN ACUTE MITOCHONDRIAL DYSFUNCTION

A model of acute cochlear mitochondrial dysfunction was generated by applying an irreversible blocker of mitochondria respiratory complex II onto the round window niche in rats (Hoya et al., 2004). In this model, severe cell loss occurred in the lateral wall spiral ligament, usually along with a profound hearing impairment (Okamoto et al., 2005). As described above, numerous inflammatory/immune-related genes were induced after the insult. Surprisingly, 35 and 60% of the genes showing 2-fold upregulation at 1 and 3 days post-3-NP administration, respectively, were inflammatory genes, including CC- and CXC-type chemokine genes (Fujioka et al., 2014). The high percentage of inflammatory genes proved that inflammatory/immune reactions occurred at the cochlear lateral wall fibrocytes. Interestingly, neutrophil receptors were never detected in the experiment, although their chemokine ligands for the recruitment of neutrophil were expressed, which is consistent with the old dogma of immune privilege. The only cell types that expressed both the receptors and ligands were those of the monocyte lineage, and this was also observed in the noise-exposed murine lateral wall (Hirose et al., 2005). Therefore, manipulating the pathways of chemokine signals, including the CCL family, could be used to develop therapeutics targeting the hearing loss caused by mitochondrial dysfunction in the inner ear.

## CONCLUSION AND FUTURE DIRECTIONS

During the past few decades, accumulating evidence from the work of several groups in the field of inner ear biology has revealed the presence of the inflammatory and immune systems in the cochlea, phenomena that had been obscured by the dogma of immune privilege. However, these findings are mainly based on models that cannot fully recapitulate clinical cases. For example, in autoimmune inner ear disorders, the fundamental mechanisms underlying the autoimmunity remain unknown, although many clinical findings over the last 40 years suggest that deafness develops from autoimmunity. Recent findings have shown the CD4+ T-cell-mediated immune surveillance in the cochlea (Solares et al., 2004), but the initial step of the autoimmunity or antigen presentation has not yet been elucidated in the cochlea, in which a drainage lymph node has not been found. Therefore, generating new models using emerging technologies and analyses based on the information reviewed in this article may explain the remaining mysteries regarding the inflammatory/immune systems in the cochlea.

## Conflict of Interest Statement

The authors declare that the research was conducted in the absence of any commercial or financial relationships that could be construed as a potential conflict of interest.
